# Retrospective clinical analysis of 320 cases of microvascular decompression for hemifacial spasm

**DOI:** 10.1097/MD.0000000000011825

**Published:** 2018-10-12

**Authors:** Zhimin Li, Jun Gao, Tianyu Wang, Yongning Li

**Affiliations:** a Department of Neurosurgery, Peking Union Medical College Hospital, Chinese Academy of Medical Sciences, Beijing; b Department of Breast Surgical Oncology, National Cancer Center/National Clinical Research Center for Cancer/Cancer Hospital & Shenzhen Hospital, Chinese Academy of Medical Sciences and Peking Union Medical College, Shenzhen, China.

**Keywords:** hemifacial spasm, microsurgery, microvascular decompression

## Abstract

To investigate effects of microvascular decompression (MVD) surgical treatment on hemifacial spasm.

A retrospective analysis of 320 adult patients (95 male cases, 29.7% and 225 female cases, 70.3%) with hemifacial spasm treated by surgery was conducted between February 2007 to June 2016, with an average age of 49.3 years and average disease course of 4.9 years. All the 320 cases of patients received MVD. After surgery, all patients were followed up for an average of 2.3 years. Surgical effects were evaluated based on the patients’ symptoms and signs. As this is just a retrospective study that does not involve any interventions, ethical approval was not necessary according to the rules of the hospital.

All patients were followed up, no death occurred. Symptom was completely disappeared in 241 cases (75.3%), 50 cases (15.6%) improved; the total effective rate of surgery was 90.9%. No obvious changes of hemifacial spasm were happened in 29 cases (9.1%). There was no deteriorated case.

MVD is one of the preferred treatments of hemifacial spasm, the intraoperative electrophysiological monitoring of abnormal muscle response signals contributes to the determination of responsible vessels and fully understanding of delayed resolution is helpful to the accuracy of surgical evaluation.

## 1 Introduction

Hemifacial spasm is a disease with a symptom of facial semi-involuntary twitching. At present, the cause of majority patients with hemifacial spasm is that the root exit zone (REZ) is compressed by the neighboring pulsatile artery thus leading to demyelination.^[1]^ Therefore, it is generally accepted that relieving the responsible vascular compression on REZ by microvascular decompression (MVD) is the most widely accepted surgical method.^[2]^ The key and difficult point of MVD is to relieve the compression, and meanwhile to protect the blood vessel and nerve functions.^[3]^ This retrospective review analyzed the clinical characteristics, treatment processes and effects of 320 cases of hemifacial spasm undergoing MVDs; and reviewed related literatures to explore the operation principles of MVD for the treatment of hemifacial spasm.

## 2 Materials and methods

### 2.1 General materials

We included 320 cases of hemifacial spasm undergoing MVDs. The disease courses ranged from 6 months to 28 years (4.9 years on average). They all underwent conservative treatments (drugs, acupuncture, and botulinum toxin injection), but the therapeutic efficacies were not satisfying; 172 cases suffered from left hemifacial spasm, and 148 were of the right side. According to the degrees of Cohen spasm standard classification,^[4]^ there were 289 cases of grade 3 and 31 cases of grade 4. Preoperative routine head magnetic resonance imaging (MRI) examinations were performed to exclude secondary hemifacial spasm caused by space-occupying lesions in the cerebellopontine angle area^[5]^; FIESTA sequence of the posterior fossa was used to understand the anatomical relationships between the facial nerve and the blood vessels nearby.

### 2.2 Methods and procedure steps

**Figure 1:**
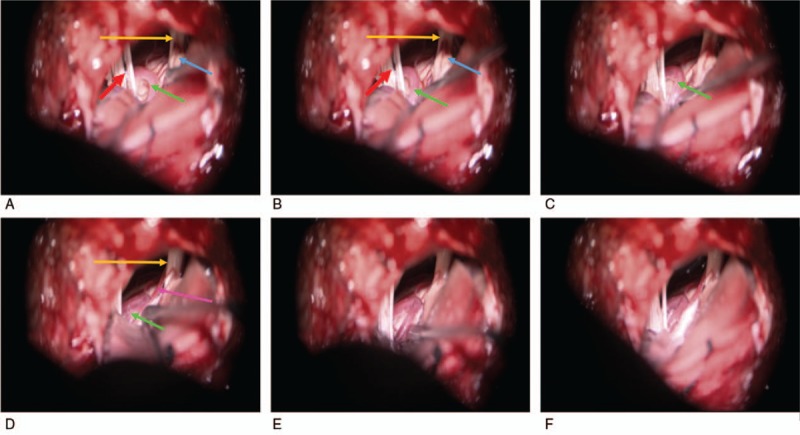
(A–F) The surgical processes are partially shown. Green arrow: anterior inferior cerebellar artery. Blue arrow: auditory nerve. Orange arrow: facial nerve. Red arrow: lower cranial nerves (vagus nerve, accessory nerve, and hypoglossal nerve). Purple arrow: labyrinthine artery.

After general anesthesia, monitoring electrodes were arranged at the ipsilateral orbicularis muscle to record abnormal changes of the muscle response like brainstem evoked potentials, which was considered as a common practice by most neurosurgeons in the world to protect the auditory complex when performing surgical manipulations in the facial-acoustic nerve. Patients were put in the lateral position with the affected side being at the top. The head was generally higher than foot for about 20° with the neck slightly bending toward the contralateral side. Local skin behind the ear was shaved. The incision was along the hairline (5 cm). Subperiosteal dissections were performed using the electric knife. Surgeons then drilled a hole at around 1 cm outside the top of mastoid vein, making a bone window about 2 cm × 2 cm in size. Lateral side was exposed until the sigmoid sinus edge. Under the microscope, the dura mater was cut curvedly and suspended, thus the subarachnoid was opened. The cerebrospinal fluid was fully released to reduce the tension of the brain tissue. The cerebellum was lifted gently from the downward outside to the upper inside. The subarachnoid was separated sharply between the auditory nerve and the posterior group of cranial nerves. After adjusting the direction of the microscope, the flocculus cerebelli was exposed and retracted upwards and inwards. Facial nerve REZ and the responsible vessel could be exposed. We then completely release the arachnoid adhesions between the responsible vessels and the REZ, free these responsible vessels, and insert a Teflon felt between these 2 layers to make the responsible vessels not contact REZ directly, and at the same time not cause obvious compressions on REZ and distortions of the responsible vessel. The surgical processes were partially shown in Fig. 1. After hemostasis, warm saline and dexamethasone were applied to rinse the surgical field. Watertight suturing of the spinal dura was performed to avoid cerebrospinal fluid leakage and secondary infections. Then we repaired the bone window with titanium plate and sutured the scalp.

### 2.3 Postoperative treatment and follow-up

Postoperatively, the consciousness and vital signs of the patients were closely monitored. During the hospital stay, doctors closely observed the patients’ relieve degree of the hemifacial spasm, facial paralysis conditions, and possible complications such as hearing loss, tinnitus, and leakage of cerebrospinal fluid. Sutures were removed at 7 days if the wound healed. The patients were asked to regularly come back to the hospital for followed-ups. The follow-up periods lasted for from 6 months to 3.5 years.

## 3 Results

### 3.1 Responsible vessels

Among the 320 patients, 95 were men and 225 were women. Their ages ranged from 23 to 73 years old. The average age was 49.3 years old. No space-occupying lesions were found in the cerebellopontine angle area by the preoperative MRI examinations. FIESTA sequence indicated that relationships between facial nerve and adjacent vessels were closed in the pool of cerebellopontine. During the surgery, 172 cases (53.8%) were found that the responsible vessels were anterior inferior cerebellar artery; 111 cases (34.7%) were posterior inferior cerebellar artery; 14 cases (4.4%) were vertebral artery; and 23 cases (7.2%) were anterior inferior cerebellar artery combined with posterior inferior cerebellar artery.

In this study, abnormal changes of the muscle response were detected in 93 patients under the electrophysiological monitoring recorded. Before the cut of the dura mater, these 93 cases were all present with abnormal muscle response waveforms in the spastic side. After the cut of the dura mater, abnormal muscle response waveforms disappeared in 7 cases (7.5%). After debriding the arachnoid adhesions in the REZ, waveforms disappeared in 12 cases (12.9%). After inserting the pad, waveforms disappeared in 67 more cases (72.0%). The abnormal waveforms still exist in 7 cases (7.5%) after surgery.

### 3.2 Surgical results

No deaths occurred in all patients; no facial paralysis occurred. Postoperative fever developed in 14 patients (4.4%), suggesting likely infection of the central nervous system. But after appropriate aggressive anti-inflammation and antibiotic treatment, they all recovered before discharges. Three cases (0.9%) had postoperative tinnitus. After neurotrophic treatment and improving microcirculation, the symptom of tinnitus disappeared. Three cases (0.9%) had mild leakage of cerebrospinal fluid postoperatively, but healed on their own.

Patients were discharged around a week after the surgery on average. Symptoms of hemifacial spasm completely disappeared in 241 cases (75.3%), improved in 50 cases (15.6%). The total effective rate of surgery was 90.9%; 29 cases (9.1%) had no obvious improvements. All patients were followed up for from 6 months to 3.5 years, symptoms of hemifacial spasm completely disappeared in 286 cases (89.4%), improved in 23 cases (7.2%), and maintained the same in 11 cases (3.4%).

## 4 Discussion

At present, 2 hypotheses have been put forward regarding the pathogenesis of hemifacial spasm: Jannetta thought that the compression on REZ by responsible vessels demyelinates the facial nerve fibers, forming false synaptic transmissions between adjacent afferent and efferent nerve fibers. Thus, the “ electric short circuit” resulted in the occurrence of hemifacial spasm.^[6,7]^ Ishikawa et al^[8]^ considered that the vascular compression increases the activities of the motor nucleus of facial nerve; normal afferent impulse becomes efferent impulse after the transhipment in the nerve nucleus, subsequently causing spasm symptoms. No matter which hypothesis is true, the “blood vessel compression on the nerve” is the anatomical basis. Therefore, the ideal treatment is to relieve the compression on the nerves by the blood vessels, which can completely relieve the oppression and meanwhile retain the nerve functions. MVD by pad compounds to separate the responsible vessels from the facial nerve root to achieve decompression effects has become the first choice for the treatment of hemifacial spasm.^[9,10]^ Microscopically assisted vascular decompression for facial spasm, which can help evaluate the ventral side of the complex in avoiding traction on the eighth nerve, has been reported to have a mortality rate of 0.2% and an overall complication rate of approximately 5% to 25% for temporary dysfunction and 2% to 10% for permanent neurologic impairment. But as for our case, we only used the microscopic techniques.^[11,12]^

### 4.1 Responsible vessels

Studies have shown that Schwann cells on the surface of facial nerve REZ have a relatively lower stress tolerance to vessel pulsatility, thus the REZ is the key place to search for the responsible vessels.^[13]^ In order to avoid possible displacement of blood vessels caused by surgical operations thus affecting identifying responsible vessels, surgeons should separate the adjacent arachnoid sharply and protect the cerebellar tissue as much as possible. First, open the arachnoid cavity between the facial nerve and the lower cranial nerves to further release of cerebrospinal fluid. Second, expose the choroid plexus at the lateral aperture of fourth ventricle, revealing the REZ of the facial nerve at the ventral side of the auditory nerve and separating the adjacent vessels. In most cases, vascular impressions on the facial nerve REZ could be seen at this time, hence the responsible vessel being confirmed.^[14]^ The surgical outcomes of MVD surgery are largely determined by whether the responsible vessel can be identified accurately. Sometimes, vessels near or parallel with the facial nerve can be easily mistaken as responsible ones, thus ignoring the real ones hidden in the pontomedullary sulcus. In the literature, most of the cases were single vessel compression, with the anterior inferior cerebellar artery being the commonest, and the posterior inferior cerebellar artery the second commonest.^[15]^ In our study, the anterior inferior cerebellar artery accounted for 53.8% of the responsible blood vessels; the posterior inferior cerebellar artery accounted for 34.7%; both of which accounted for more than 85% of the responsible vessels. Of note, compression from multiple vessels should be considered, where the main responsible vessels are frequently located on the deep surface of the vascular plexus. We found in this study, compressions on REZ from the anterior inferior cerebellar artery and posterior inferior cerebellar artery and their vascular loops accounted for 7.2% of all cases. Although pulsating compressions are mostly caused by arteries, there was a study suggesting that veins could also be the source of compression in addition to arteries.^[16]^ In our study, symptoms of 3 patients with more than 1 year of hemifacial spasm were not significantly improved. In these cases, all possible arteries around the REZ were carefully separated during the surgery, but veins adjacent to the REZ were not operated on, which might be the reason for which the postsurgical symptom improvements were not obvious. Yu et al proposed that the veins adjacent to the facial nerve REZ should also be taken seriously; but if the responsible veins were too large, surgeons should not deliberately separate them in case of uncontrolled bleeding or brain stem infarction.^[17]^

### 4.2 Electrophysiological monitoring

For patients with hemifacial spasm, an abnormal muscle response (AMR) can be elicited by electrical stimulation of a branch of the facial nerve and recorded from muscles innervated by other branches of the facial nerve.^[18]^ A large number of the literature reported that after releasing the pressure on nerve root by MVD, the majority of patients’ symptoms of AMR disappeared; after restoring the compression, AMR re-emerged.^[19–22]^ Therefore, electrophysiological monitoring of AMR during the MVD can help confirm the responsible vessels, thus further improving the surgical effects. Mooij et al^[23]^ performed 74 cases of MVDs under AMR monitoring, at the cure rate over 92%. Wang et al's cure rate of 40 cases under AMR monitoring was 94.7%.^[24]^

In our study, preoperational AMR was recorded in 93 cases. When cutting the dura mater or the arachnoid between the surface of auditory nerve and the posterior cranial nerves, the abnormal waveforms disappeared in 19 cases (20.4%); after separating the responsible vessels in REZ and putting appropriate pads, the abnormal waveforms disappeared in 67 cases (72.0%). The possible explanation of this phenomenon was that when the dura mater or the arachnoid was cut, the release of cerebrospinal fluid changed the hydromechanics of cerebrospinal fluid in the cerebellopontine angle area; the local pressure thus decreased, which relived the compression to some extent. Only by completely decompressing the REZ, could most AMRs disappeared. It is noted that during the surgery, it is a sufficient condition rather than a necessary condition that the disappearance of AMRs suggesting successful surgical results. Kong et al^[25]^ reported that 33 cases (12.5%) in 263 patients still had AMRs after the decompression. Li et al^[26]^ reported that the proportion of postsurgical AMR existence was around 9.6%. We hypothesize that it would take a longer time for Schwann cells to proliferate to a normal level, for the nerve fiber myelin sheath to get repaired, and for the motor nucleus of facial nerve to be reactivated, if the responsible vessel continued compressing the REZ which would cause a severe demyelinating neuropathy and continuous high activities of the motor nucleus of facial nerve, even if the responsible vessel had been separated with the nerve root.

### 4.3 Surgical effects

The surgical efficiency (including complete disappearance and significant remission of hemifacial spasm symptoms) was estimated at 92.9% to 98%.^[1,3]^ Yuan et al^[27]^ followed up 1200 cases of surgically treated hemifacial spasm patients for 2 to 10 years. The symptoms of 94.3% of the cases disappeared or significant relieved. Yin et al^[28]^ retrospectively analyzed 833 cases and the total curative effectiveness rate was 92.9%. Jin et al^[29]^ followed up 1010 cases of surgically treated hemifacial spasm patients, 98.0% getting significantly relieved 1 year postoperatively. Lee et al^[30]^ followed up 2027 patients, among which 1841 patients’ (90.8%) symptoms disappeared or significantly relieved, 113 patients’ (5.6%) symptoms improved compared with before. The total effective rate was 96.4%.

After the operation, in most patients, the symptoms will disappear; while symptoms of a small group of patients will be relieved gradually until disappearing completely rather than disappear immediately after surgery—a phenomenon called “delayed resolution.”^[31]^ It was reported that the rate of delayed resolution could be up to 36.3%.^[31]^ The reason may be that the complete recovery of facial nerve root demyelination and the reactivation of the facial nerve motor nucleus needs months or even years.^[31,32]^ Chang et al^[33]^ considered that despite the full decompression, facial nerve root may still suffer from pulsatile impact from the cerebrospinal fluid pressure, resulting in extended length of recovery. Ray et al^[14]^ found patients with deeper compression impression at REZ generally need longer time to recovery, thus believing that the delayed recovery was related with the degrees of the nerve root damage.

### 4.4 Strengths and limitations

It is generally accepted that relieving the responsible vascular compression to REZ by MVD is the most widely accepted surgical method. The key and difficult point of MVD is to relieve the pressure, but also to retain the blood vessel and nerve function. Our retrospective review analyzed the clinical characteristics, treatment processes, and effects of 320 cases of hemifacial spasm undergoing MVDs in detail. The sample size is relatively larger than most previous ones. We conclude that MVD surgery is one of the preferred treatments of hemifacial spasm, the intraoperative electrophysiological monitoring of abnormal muscle response signals contributes to the determination of responsible vessels and fully understanding of delayed resolution is helpful to the accuracy of surgical evaluation. Limitations of our study include: because the present study is a retrospective and descriptive one, there were no relevant analytical statistics provided. Data were only presented in a descriptive manner. In future studies, analytical statistics for the follow-up results of different surgical procedures or therapeutic method for hemifacial spasms are worth performing. The follow-up periods only lasted for from 6 months to 3.5 years. More follow-up time is expected.

## Acknowledgments

The authors would like to thank our colleagues from the Department of Neurosurgery, Peking Union Medical College Hospital, Chinese Academy of Medical Sciences, and Peking Union Medical College.
